# Proactive Model of Remote Interaction for Treating Post-COVID Anxiety and Depression in the Context of War in Ukraine

**DOI:** 10.1192/j.eurpsy.2025.463

**Published:** 2025-08-26

**Authors:** O. O. Khaustova, A. O. Burdeinyi, O. S. Chaban

**Affiliations:** 1 Department of Medical Psychology- Psychosomatic Medicine and Psychotherapy; 2 Bogomolets National Medical University, Kyiv, Ukraine

## Abstract

**Introduction:**

Remote interaction offers new opportunities for medical-psychological rehabilitation, providing access when in-person consultations are limited. The proactive model of psychosomatic medicine, focusing on prevention and can to enable continuous monitoring and active patient engagement, crucial for those affected by post-COVID syndrome and war.

**Objectives:**

Analyze the effectiveness of a medical-psychological rehabilitation program for anxiety and depressive post-COVID disorders, as well as post-traumatic symptoms resulting from war, in the context of remote interaction.

**Methods:**

The study sample consisted of 240 individuals with anxiety and depressive post-COVID disorders. The tools used in the study included the PHQ-9, GAD-7, and PCL-5.

**Results:**

By day 63, according to the PHQ-9, depression in the study group decreased to 6.942±5.073, while in the control group it remained at 15.567±6.540 (p < 0.001; t = 11.437). According to the GAD-7, anxiety reduced to 3.991±3.589 in the study group, whereas in the control group it remained higher at 12.966±3.980 (p < 0.001; t = 18.355). According to the PCL-5, PTSD symptoms decreased to 13.295±8.727 in the study group, while in the control group they remained high at 29.177±13.541 (p < 0.001; t = 10.836).

**Conclusions:**

The study results indicate that the medical-psychological rehabilitation program, delivered through remote interaction, effectively reduces symptoms of depression, anxiety, and post-traumatic stress in individuals with post-COVID disorders amid wartime conditions.

**Disclosure of Interest:**

None Declared

